# Arbovirus Epidemics as Global Health Imperative, Africa, 2023

**DOI:** 10.3201/eid3102.240754

**Published:** 2025-02

**Authors:** Salifou T. Bangoura, Alpha-Kabinet Keita, Maladho Diaby, Sidikiba Sidibé, Frederic Le-Marcis, Saidouba C. Camara, Stéphanie Maltais, Kadio J.J.O. Kadio, Eric D’Ortenzio, Alioune Camara, Eric Delaporte, Alexandre Delamou, Philippe Vanhems, Michèle Ottmann, Nagham Khanafer, Abdoulaye Touré

**Affiliations:** Gamal Abdel Nasser University, Conakry, Guinea (S.T. Bangoura, A.-K. Keita, M. Diaby, S. Sidibé, K.J.J.O. Kadio, A. Camara, A. Delamou, A. Touré); Centre de Recherche et de Formation en Infectiologie de Guinée, Conakry (S.T. Bangoura, A.-K. Keita, M. Diaby, F. Le-Marcis, S.C. Camara, K.J.J.O. Kadio, A. Camara, A. Touré); TransVIHMI, Université de Montpellier-INSERM-IRD, Montpellier, France (A.-K. Keita, F. Le-Marcis, E. Delaporte); Ecole Normale Supérieure de Lyon, Lyon, France (F. Le-Marcis); University of Ottawa School of International Development and Global Studies, Ottawa, Ontario, Canada (S. Maltais); ANRS Maladies infectieuses émergentes, Inserm, Paris, France (E. D’Ortenzio); AP-HP, Hôpital Bichat, Service de maladies infectieuses et tropicales, Paris (E. D’Ortenzio); African Centre of Excellence in the Prevention and Control of Communicable Diseases, Gamal Abdel Nasser University, Conakry (A. Delamou); National Center for Training and Research in Rural Health of Maferinyah, Forécariah, Guinea (A. Delamou); Centre International de Recherche en Infectiologie, Inserm, Lyon (P. Vanhems, M. Ottmann, N. Khanafer); Infection Control Unit, Hôpital Edouard Herriot, Hospices Civils de Lyon, Lyon (P. Vanhems, N. Khanafer)

**Keywords:** viruses, vector-borne infections, Africa, arboviruses, epidemic, global health

## Abstract

Arboviruses represent a major cause of illness in Africa and have the potential to trigger widespread epidemics. We present data on arbovirus epidemics in Africa in 2023 and demonstrate the need for global public health authorities to intensify efforts in the surveillance and control of arbovirus diseases. Data were collected from the World Health Organization Weekly Bulletin on Outbreaks and Other Emergencies, Africa Centers for Disease Control and Prevention Weekly Event Based Surveillance Report, and other online sources. In 2023, a total of 7 arboviruses were responsible for 29 outbreaks across 25 countries in Africa, 22 of which occurred in West Africa; the outbreaks resulted in 19,569 confirmed cases and 820 deaths. Arbovirus epidemics in Africa pose a threat not only to public health within the continent but also globally, underscoring the urgent need for substantial investment in arbovirus surveillance, research, and preparedness capacities in Africa to prevent and respond to health crises effectively.

Arthropodborne viral diseases represent a major global health challenge because of their capacity to cause explosive outbreaks and induce severe, potentially life-threatening clinical conditions, including encephalopathy, meningoencephalitis, myelitis, and symptoms of Guillain-Barré syndrome (*1*,*2*). Dengue virus (DENV) is the most prevalent arbovirus; >7.6 million cases had been reported to the World Health Organization (WHO) as of April 30, 2024, including 3.4 million confirmed cases, >16,000 severe cases, and >3,000 deaths (*3*), imposing substantial economic burdens on many tropical and subtropical countries (*4*,*5*).

The frequency and scale of outbreaks caused by these arboviruses, particularly those transmitted by *Aedes* mosquitoes, are rising globally, driven by the intersection of ecologic, economic, and social factors (*6*,*7*). In response, the WHO launched the Global Arbovirus Initiative to “raise the global alarm on the risk epidemics of arboviruses and the potential risk of pandemics” (*6*,*8*). This initiative focuses on risk monitoring; pandemic prevention, preparedness, detection, and response; and the development of a coalition of partners (*6*). However, the capacity of countries in Africa to respond to arboviral threats is a concern, because most lack the necessary infrastructure and resources (laboratory equipment, trained personnel, and funding) to conduct adequate surveillance of arthropods and the viruses they transmit, whereas performing diagnostic tests to differentiate among viruses presents substantial challenges (*9*–*12*). As a result, most arbovirus infections go undiagnosed until epidemics emerge, causing severe health, social, and economic consequences (*13*–*15*). Furthermore, seroprevalence studies indicate that both endemic and epidemic transmission of arboviruses occurs regularly across Africa (*2*,*16*–*18*). In this context, where arbovirus infections circulate frequently in low- and middle-income countries—particularly in Africa, where health needs remain unmet—concern persists regarding the potential export of these viruses to previously unaffected regions, driven by global demographic, societal, and environmental trends of the 21st Century (*19*,*20*). A recent example is Zika virus (ZIKV), which swiftly transitioned from obscurity to a WHO Public Health Emergency of International Concern (*21*,*22*). That potential underscores the necessity for substantial investment in the arbovirus surveillance, research, and preparedness capacities of countries in Africa to effectively prevent and respond to future public health threats. In 2023, multiple arbovirus epidemics occurred in Africa, frequently occurring simultaneously and transcending borders (*7*,*23*,*24*). In this review, we present data on arbovirus epidemics reported in Africa in 2023, emphasizing the need for global public health authorities to take steps toward an equitable distribution of health efforts and resources that could enhance both local and global health security.

## Methods

We compiled a database of arbovirus-related human epidemics reported in Africa during 2023, sourcing information from the WHO Weekly Bulletin on Outbreaks and Other Emergencies and the Africa Centers for Disease Control and Prevention Weekly Event-Based Surveillance Report. In addition, we consulted various online resources related to infectious diseases, including ProMED-mail (http://www.promedmail.org) and the International Society for Infectious Diseases (http://www.isid.org). For cases reported on ProMED-mail, we verified the source of information, specifically checking whether information was in the form of an official statement from public health authorities. We used a standardized form to extract and enter the data into an Excel spreadsheet (Microsoft, https://www.microsoft.com). The dataset encompassed the country where the epidemic occurred, the type of arbovirus, the reporting date of the first cases, the number of suspected cases, the number of confirmed cases, the number of deaths, and the data sources. The country's notification date was used to remove duplicates. We conducted a descriptive analysis of the data to summarize the findings. We categorized arbovirus infection cases into 2 groups: suspected cases, which included all reports lacking laboratory confirmation, and confirmed cases, which consisted of all reports with biological verification through PCR or IgM ELISA.

## Results

In total, 29 arboviral outbreaks were reported across 25 countries in Africa in 2023; of those, 22 occurred in West Africa (Figure). Seven distinct arboviruses were responsible for the outbreaks: DENV in 17 countries; yellow fever virus (YFV) in 9 countries; chikungunya virus (CHIKV) in 4 countries; Crimean-Congo hemorrhagic fever virus (CCHFV), Rift Valley fever virus (RVFV), and West Nile virus (WNV) each in 3 countries; and ZIKV in 2 countries. Senegal recorded the highest number of outbreaks linked to arboviruses (DENV, CHIKV, ZIKV, YFV, CCHFV, and WNV), followed by Namibia (CHIKV, CCHFV, and WNV) and Mali (DENV, CHIKV, and ZIKV) (Figure).

**Figure:**
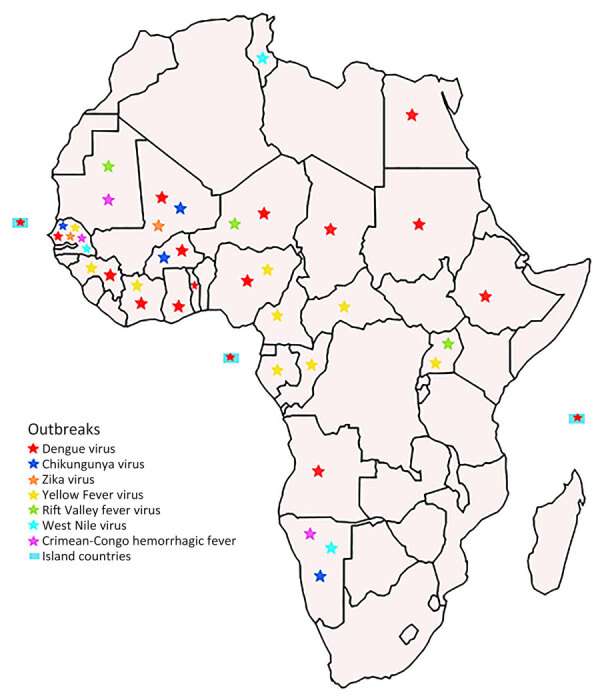
Geographic distribution of confirmed arbovirus cases reported across Africa, 2023.

In 2023, a total of 19,569 confirmed cases and 820 deaths were reported. Most infections were attributed to DENV, which accounted for 18,697 confirmed cases and 771 deaths. Burkina Faso faced an unprecedented epidemic, reporting 17,125 confirmed DENV cases and 688 deaths. Mali followed with 808 confirmed cases and 34 deaths, then Ethiopia with 272 cases and 17 deaths and Senegal with 254 confirmed cases but no fatalities. Nine countries (Cameroon, Côte d'Ivoire, Central African Republic, Gabon, Guinea, Nigeria, Republic of Congo, Senegal, and Uganda) reported a total of 104 confirmed yellow fever cases, resulting in 39 deaths. The highest number of cases was recorded in Cameroon (49 cases, 6 deaths), Nigeria (21 cases, 21 deaths), Congo (15 cases, 2 deaths), and the Central African Republic (13 cases, 6 deaths). CHIKV infections were documented in 4 countries, with most cases reported in Burkina Faso (351 confirmed cases, 1 death) and Senegal (338 confirmed cases, no deaths) (Table).

**Table:** Distribution of arbovirus cases in humans, Africa, 2023*

Type of arbovirus	Country	No. suspected cases	No. confirmed cases	No. deaths
Dengue	Burkina Faso	21,4536	17,125	688
	Mali	5,369	808	34
	Ethiopia	20,486	272	17
	Senegal	ND	254	0
	Chad	2,013	72	1
	Mauritius	222	40	0
	São Tomé and Príncipe	ND	31	22
	Egypt	500	31	0
	Côte d'Ivoire	85	22	0
	Nigeria	72	14	0
	Ghana	18	9	0
	Togo	ND	8	1
	Cabo Verde	58	7	0
	Angola	ND	3	0
	Guinea	5	1	1
	Niger	148	0	0
	Sudan	1,664	ND	7
	Total	245,176	18,697	771
Yellow fever	Cameroon	10	49	6
	Nigeria	1,798	21	21
	Congo	374	15	2
	Central African Republic	336	13	6
	Guinea	262	3	4
	Senegal	ND	2	0
	Côte d'Ivoire	ND	1	0
	Gabon	119	ND	0
	Uganda	12	ND	0
	Total	2,911	104	39
Chikungunya	Burkina Faso	234	351	1
	Senegal	ND	338	0
	Namibia	ND	1	0
	Mali	4	1	0
	Total	238	691	1
Crimean Congo hemorrhagic fever	Senegal	ND	8	2
	Mauritania	ND	3	0
	Namibia	ND	1	1
	Total	ND	12	3
Rift Valley Fever	Uganda	20	27	4
	Niger	ND	1	1
	Mauritania	ND	1	0
	Total	20	29	5
West Nile	Tunisia	5	10	1
	Namibia	ND	1	0
	Senegal	ND	1	0
	Total	*5*	12	1
Zika	Mali	ND	22	0
	Senegal	ND	2	0
	Total	ND	24	0

*ND, not defined

Confirmed cases of CCHFV infection were documented in Senegal (8 cases, 2 fatalities), Mauritania (3 cases, no fatalities), and Namibia (1 fatal case). RVFV infection also led to several confirmed cases and deaths, notably in Uganda (27 cases, 4 deaths), whereas isolated cases were reported in both Niger and Mauritania. Tunisia registered the highest number of WNV infections, with 10 confirmed cases. Mali and Senegal together reported 24 confirmed cases of ZIKV infection, with no associated fatalities (Table).

## Discussion

The repeated and concurrent arbovirus epidemics across Africa raise concerns about the effectiveness and sustainability of current control and surveillance strategies in the region. This situation highlights the pressing need for comprehensive investments in national health systems to enhance clinical and biologic surveillance and to strengthen control and prevention efforts against these diseases.

West Africa has been disproportionately impacted by multiple, concurrent arbovirus outbreaks. This finding aligns with previous studies (*10*,*25*), raising concerns about the region's capacity to conduct effective surveillance of arboviruses and their vectors, and to detect, prevent, and respond to epidemics. The conclusions of the survey assessing the capacity of health systems to prevent, detect, and respond to epidemics of arbovirus diseases in the 47 countries of the WHO Regional Office for Africa revealed that the West Africa region had inadequate capacity for virologic surveillance and routine vector surveillance and control (*12*). Moreover, the region’s diverse vegetation, favorable climate, and presence of both wild and domestic vertebrate hosts create an ideal environment for vectors (*1*,*10*,*26*,*27*). Furthermore, models predicting the global distribution of arbovirus diseases highlight West Africa as a high-risk area because of its heavy rainfall and optimal temperature conditions for both endemic and epidemic transmission (*4*,*28*). As a consequence, adopting innovative diagnostic and surveillance methods, such as genomic sequencing, will be critical to enhance early detection of both endemic and emerging arboviruses, assess viral strains’ epidemic potential, evaluate intervention effectiveness, and map transmission networks across humans, animals, and vectors (*20*,*29*). For example, in Nigeria, metagenomic sequencing was used to identify an ongoing yellow fever outbreak and its etiology and inform real-time public health actions, resulting in accurate and timely disease management and control (*30*). The ability of genomic sequencing to rapidly identify and characterize reemerging viruses offers a valuable opportunity for enhancing public health surveillance. Furthermore, fostering cross-country collaboration and networking is crucial for harmonizing laboratory diagnostics and strengthening epidemic preparedness. Without such coordinated efforts, isolated interventions might be inadequate or entirely ineffective (*9*,*11*).

DENV was the most frequently reported infection; major epidemics occurred in Burkina Faso, Mali, Sudan, and Senegal. DENV is the most prevalent arbovirus globally; Africa accounts for 16% of all infections, positioning it as the second most affected region after Asia (*31*,*32*). Since the 1960s, DENV has remained a leading cause of illness across all African subregions (*32*); outbreaks have been documented in numerous countries, such as Tanzania (*33*–*35*), Mozambique (*36*), Ghana (*37*,*38*), Sudan (*13*), Benin (*39*), Burkina Faso (*40*), and Côte d’Ivoire (*41*). DENV is not only of considerable medical importance but also imposes a substantial economic burden, estimated in billions of dollars annually (*42*,*43*).

YFV remains a public health challenge in Africa, despite the availability of a highly effective vaccine that induces strong and long-lasting immunity (*44*,*45*). Numerous cases, some fatal, have been documented across the continent. The persistence of yellow fever in Africa is largely attributable to insufficient vaccination coverage; the disease is most prevalent in tropical sub-Saharan Africa, where it is preventable primarily through vaccination (*46*). According to WHO/United Nations Children’s Fund estimates on national immunization coverage, regional coverage stood at only 48% in 2021, far below the 80% threshold needed to achieve herd immunity against yellow fever (*47*,*48*). This shortfall was likely caused by the concurrent outbreaks of COVID-19, Ebola virus disease, measles, and cholera, which disrupted routine vaccination services, restricted movement, and reduced healthcare access. Therefore, countries must take steps to strengthen national immunization programs, alongside implementing supplementary vaccination campaigns in high-risk areas. Those campaigns should include catch-up campaigns targeting vulnerable populations to sustain high vaccination coverage. Such efforts must be coupled with implementation research to ensure equitable vaccine access. Given that implementation research is often context-specific, bolstering local capacity to identify challenges and barriers to adequate coverage would benefit all nations (*49*).

The findings of this study contribute valuable insights into the ongoing circulation of arboviruses in Africa and underscore the critical need to enhance surveillance efforts for these viruses in both vectors and human populations. They also reinforce the importance of integrated strategies for epidemic prevention and response, including vector control, expanding vaccination coverage, promoting the use of mosquito nets, and implementing education and communication initiatives aimed at fostering sustainable behavioral changes.

The first limitation of this study is that data came mainly from official public health sources, but numbers could be underestimated because of the weakness of healthcare systems in surveillance and the clinical overlap between arbovirus infections and endemic diseases such as malaria, which limits access to diagnostic testing. In addition, healthcare workers’ limited knowledge about arboviruses could lead to misdiagnosis and affect the data, as demonstrated by a recent study in public health facilities in Conakry, Guinea, where only 1% of healthcare workers had adequate knowledge of arboviruses (*50*). Moreover, this study examined data collected over a year, but it did not analyze long-term trends in arbovirus epidemics. Furthermore, the data used in this study were sourced from open-access platforms. As a result, their completeness and accuracy depend on the availability of reports from the surveillance systems of each country. Therefore, caution must be exercised in interpreting the findings, and comparisons between countries should be avoided. Repeated serologic surveys could serve as a valuable population-based surveillance tool to track trends in arbovirus infections, offering vital information for shaping public health policies, guiding vaccine deployment strategies, and identifying regions at elevated risk for future outbreaks (*51*).

The primary goal of global health, as promoted by international public health organizations, is to ensure equitable distribution of health resources and knowledge across all regions, including Africa (*52*). However, funding is often tied to specific priorities and agendas, making research programs in Africa vulnerable to fluctuations in financial support and the shifting interests of northern partners, leaving critical areas of research underfunded (*53*).

Today, the threat posed by arboviruses to global health and economic security is widely acknowledged (*54*). Not only are arbovirus outbreaks increasing, but the risk for a pandemic looms (*54*). This concern was emphasized by the WHO’s Director for Pandemic and Epidemic Diseases during the launch of the Global Arbovirus Initiative, who noted that “the next pandemic could very likely be caused by a new arbovirus, and we already have some signals that the risk is increasing” (*6*,*8*).

Despite substantial global financial investments in vaccines, access and usage remain major challenges in Africa. According to the list of vaccines authorized by the US Food and Drug Administration and the WHO, vaccines exist for DENV, YFV, tick-borne encephalitis virus, Japanese encephalitis virus, and CHIKV (*55*,*56*). Yet, the YFV vaccine is the only one currently available in Africa. Africa is one of the regions most affected by these arboviruses and has limited resources for their prevention, monitoring, and control, but those diseases continue to be neglected on the continent, despite receiving substantial attention and resources in other regions (*57*–*60*). 

Making equitable and sustainable investments in health and economic systems and infrastructure is imperative to enhance the quality and efficiency of health services in Africa, effectively preventing future global health emergencies. This effort involves strengthening laboratory and medical staff capacities by providing access to innovative and rapid diagnostic techniques (such as serologic and molecular multiplex tests, commercial kits for the rapid detection of certain arboviruses), particularly in areas endemic for arboviruses representing major health risks. Increasing access to testing could considerably improve the early diagnosis of cases, the speed of epidemic management, and consequently, the treatment of patients (*61*). Continued support from countries in Africa is needed to develop and implement surveillance and control programs for arbovirus vectors adapted to local contexts; to better understand the genetic diversity of species, their distribution, and their abundance; and to develop more effective and sustainable tools and strategies for the control of arboviruses and vectors. Furthermore, funding research in Africa on known and new arboviruses is essential for understanding their ecologies and the interactions between the components of vector systems (vectors, hosts, pathogens, and environment) and the evolution of these interactions, including the mechanisms of adaptation to new environments. Such investments represent a long-term commitment to building public health capacity and global health security (*62*), and the development of research agendas for global health initiatives should take into account the national needs and priorities of countries, in consultation with local stakeholders in the intervention contexts (*52*). Adopting a holistic, multi-sectoral, and collaborative approach that addresses the unique challenges of combating arboviruses in Africa is also essential. This collaborative effort will not only mitigate immediate epidemics but will also establish a resilient and adaptive framework for addressing future outbreaks (*63*).

## Conclusions

This study demonstrates that arboviruses are actively circulating in Africa, resulting in severe epidemics and fatalities across various regions. Currently, no specific treatments are available, and access to effective vaccines remains limited. The primary strategies for effectively combating arboviruses include robust surveillance and control of vector populations, alongside research initiatives aimed at enhancing vaccine coverage and accessibility. In addition, strengthening personal and community preventive measures such as the use of mosquito nets and the management of water-containing containers is essential. Those efforts are anticipated to yield substantial benefits within the framework of global health.

Sustained support for arbovirus surveillance and research activities in Africa is critical not only for pandemic preparedness but also for enhancing overall health resilience. Furthermore, regional and cross-border collaboration should be established in alignment with international health regulations to develop adequate capacities for preventing arbovirus diseases. Forming global alliances is essential to consolidate resources and strengthen capabilities related to arbovirus surveillance and response.
